# Porcine respiratory disease complex: Dynamics of polymicrobial infections and management strategies after the introduction of the African swine fever

**DOI:** 10.3389/fvets.2022.1048861

**Published:** 2022-11-25

**Authors:** Pornchalit Assavacheep, Roongroje Thanawongnuwech

**Affiliations:** ^1^Department of Veterinary Medicine, Faculty of Veterinary Science, Chulalongkorn University, Bangkok, Thailand; ^2^Department of Pathology, Faculty of Veterinary Science, Chulalongkorn University, Bangkok, Thailand; ^3^Faculty of Veterinary Science, Center of Emerging and Re-emerging Infectious Diseases in Animals, Chulalongkorn University, Bangkok, Thailand

**Keywords:** African swine fever, infection, interaction, management, pigs, porcine respiratory disease complex, repopulation

## Abstract

A few decades ago, porcine respiratory disease complex (PRDC) exerted a major economic impact on the global swine industry, particularly due to the adoption of intensive farming by the latter during the 1980's. Since then, the emerging of porcine reproductive and respiratory syndrome virus (PRRSV) and of porcine circovirus type 2 (PCV2) as major immunosuppressive viruses led to an interaction with other endemic pathogens (e.g., *Mycoplasma hyopneumoniae, Actinobacillus pleuropneumoniae, Streptococcus suis, etc*.) in swine farms, thereby exacerbating the endemic clinical diseases. We herein, review and discuss various dynamic polymicrobial infections among selected swine pathogens. Traditional biosecurity management strategies through multisite production, parity segregation, batch production, the adoption of all-in all-out production systems, specific vaccination and medication protocols for the prevention and control (or even eradication) of swine diseases are also recommended. After the introduction of the African swine fever (ASF), particularly in Asian countries, new normal management strategies minimizing pig contact by employing automatic feeding systems, artificial intelligence, and robotic farming and reducing the numbers of vaccines are suggested. Re-emergence of existing swine pathogens such as PRRSV or PCV2, or elimination of some pathogens may occur after the ASF-induced depopulation. ASF-associated repopulating strategies are, therefore, essential for the establishment of food security. The “repopulate swine farm” policy and the strict biosecurity management (without the use of ASF vaccines) are, herein, discussed for the sustainable management of small-to-medium pig farms, as these happen to be the most potential sources of an ASF re-occurrence. Finally, the ASF disruption has caused the swine industry to rapidly transform itself. Artificial intelligence and smart farming have gained tremendous attention as promising tools capable of resolving challenges in intensive swine farming and enhancing the farms' productivity and efficiency without compromising the strict biosecurity required during the ongoing ASF era.

## Introduction

Porcine respiratory disease complex (PRDC) is a multifactorial syndrome affecting the respiratory system of pigs in the swine industry worldwide (1, 2). Environmental factors and management practices can trigger PRDC pathogens to cause severe health problems in postweaning and weaning-to-finishing pigs. The causative agents of PRDC include primary pathogens such as porcine reproductive and respiratory syndrome virus (PRRSV), porcine circovirus type 2 (PCV2), swine influenza A virus (IAV), Aujeszky's disease virus (ADV), as well as *Mycoplasma hyopneumoniae* (Mhp) and *Actinobacillus pleuropneumoniae* (App). They can also include secondary pathogens such as *Pasteurella multocida* (Pm), *Bordetella bronchiseptica* (Bb), *Gläesserella* (*Haemophilus*) *parasuis* (Gps), and *Streptococcus suis* (Ss) (1, 2). Dynamic polymicrobial infections among the PRDC pathogens are known to exert significant effects on different clinical outcome models of coinfection or superinfection (2–6). In the period prior to the outbreak of the African swine fever (ASF), the PRDC situation in many countries appeared to be a major problem for the pig production industry (7). This situation has changed due to the outbreak of ASF in Europe and Asia.

Several factors can influence on the PRDC prevalence, including a remarkably lower number of the pig population in the affected area, the closing of pig farms due to ASF depopulation, and an improvement of the employed biosecurity program. The worldwide epidemic outbreak of the severe acute respiratory syndrome coronavirus 2 (SARS-CoV-2) also affected pig producers, both directly and indirectly (8–12). As a result, biosecurity measures have been widely improved in livestock animal production. The concepts of biosecurity practice are mainly divided into two types: those implementing external and internal measures (13). External biosecurity measures include the adoption of physical barriers so as to inhibit the introduction of disease through replacements, quarantines, and controlled use of semen, people, or vehicles, restriction of animal transportation, avoidance of the risk of disease transmission from the neighborhood as well as from the feed and the water. Common internal biosecurity measures are focused on avoiding the spreading of disease within the pig farm and involved actions related to management, personnel, facilities as well as cleaning and disinfection.

## ASF overview

ASF is a major health-threatening disease causing high lethality in domestic pigs and wild boars (14). The etiological agent is African swine fever virus (ASFV), a large double-stranded enveloped DNA virus belonging to the Family *Asfarviridae*. The ASFV genome size of approximately 170–190 kb encodes many proteins (15). Based on the partial nucleotide sequence of the capsid gene, at least 24 genotypes are established (16). Spreading of ASF has been reported in many countries in Africa, Europe and Asia (17, 18). As part of the ASF prevention strategy, biosecurity measures are considered as the most effective approach since the research and development of the ASF vaccines have not successfully met expectations. Therefore, the improvement of the farm's biosecurity should be emphasized as part of a strict management compliance approach to zero ASFV exposure, including disinfection, multisite production, parity segregation, batch production, and the all-in all-out (AIAO) production systems (Table 1).

**Table 1:** List of internal biosecurity measures essential to zero ASFV exposure.

**Biosecurity**	**List**
AIAO production system	- AIAO and complete empty of farrowing sows and fattening houses - Move all sows of each batch to farrowing units in one occasion
Separation	Housing - Partitioning house for breeders and fattening pigs - Closed house - Low pig density - Separate hospital unit
	Pig flow - Batch production - Parity segregation (P0-1) - Wean-to-finish production - Multi-site production - Separate every 3–5 pregnant sow pen and food trough
ASF monitoring	- Quarantine and acclimatization of replacement animals - Sentinel pigs before repopulation in all houses - Routine real-time PCR of all batches of sows and weaned/fattening pigs

Learning from the biosecurity used in pig farms in China, several measures including the avoidance of the introduction of pigs or semen from ASF-affected regions, the prohibition of pork or related products from outside the farm, the banning of protein products of porcine origin in feed, the admittance of no visitors from ASF-epidemic regions, the cleaning and the disinfection of trucks before and after use for pig transportation, the adoption of personnel quarantine, and the systematic elimination of ASF vectors are implemented (19). Notably, tooth extraction is an alternative control measure based on the “test and removal” method adopted for targeted depopulation in the affected herd in China and Vietnam (20–22). The protocols used for the affected sow herds include the removal of sick sows and of two sows on each side of the index sows to stop ASF spreading within the affected area. The polymerase chain reaction (PCR) test results of ASF-suspected sows must be reported to the farms as soon as possible in order to promptly remove these affected sows. If the sows were found negative, retesting must be done on the next day (23). However, based on the Vietnamese investigation demonstrated that the use of ASF tooth extraction was not sufficient to eliminate the disease, if the infected sows without clinical signs escaped from the detection (24). Recent information revealed that the ASF surviving pigs are the potential source of reinfection, as remaining carriers of the ASFV in their organ tissues, but not in serum. In order to be able to better prevent a recurrent outbreak, the infected but surviving sows should not be further used for pig production, as the probability of a stress induced ASFV recurrence in the long-term surviving pigs is expected (25).

Along with the tooth extraction measurement, the partitioning of the production unit has also been implemented as a method for the reduction of the disease transmission among units, while a targeted pig removal from only the ASF-affected unit led to lower losses of production with satisfactory results in the earlier ASF-detection farms (21). Indeed, the structure of partitioning in the individual farm level composes of three main parts as (i) the external and internal biosecurity measures to mitigate the opportunity to ASF introduction and maintain the separation of subpopulations, (ii) the cost-effective of on-farm ASF surveillance to strengthen early detection and reporting, and (iii) the reaction plans at the production unit level, including culling of affected subpopulations, and demonstration of freedom from disease on the remaining ones. The overall outcome of partitioning in an ASF outbreak case is to avoid full farm depopulation (21).

After the ASF outbreaks, the “farm rebirth” policy in China and Vietnam–referring especially to small-to-medium pig farms–has been a topic of interest as a response to the re-occurrence of ASF in the local pig industry. In order to avoid the ASF re-occurrence, the Chinese government and the related authorities have launched a program of financial support for a culling subsidy of livestock in the infected farms, and have established the criteria for the repopulation or rebirth of these farms (26). This must be along with the application of a strict biosecurity management of these pig farms with particular emphasis on the control of vehicles, personnel / visitors, fomites, vectors / pests, food and water supply for humans and pigs, infrastructure renovation / disinfection, the application of closed systems for the pig houses, changes of the environment, the distribution of sentinel pigs, the replacement of gilts / boars, and the employment of batch production with an AIAO system for the farrowing unit. As part of these measures, an essential management strategy aiming to reduce the human-to-pig and the pig-to-pig contact should be planned so as to fit the farm conditions and provide automatic feeding and automatic pig grouping. As a result, artificial intelligence (AI) and smart farming have gained tremendous attention in order to resolve such challenges in intensive swine farming, and to enhance the farm productivity and efficiency without compromising the strict biosecurity required during the ASF era. Automatic feeding systems, AI, and robotic farming have been exploited in order to reduce the opportunity of an ASFV transmission. The application of AI-guided smart farming also provides information that can be used for diagnostic purposes, growth and productivity monitoring, as well as environmental management (27–29). In the repopulate farms in the designated location, the “test and removal” policy and the herd biosecurity should be strictly applied. The new normal management practice of those repopulate farms located in the farm zoning designations should adopt a partitioning strategy. Partitioning is not only used for individual farms, but can also be applied for different stakeholders, since it is a public-private partnership network in which all sectors should work together in order to reduce the risk of ASF. A combination of all available biosecurity strategies is necessary in order to maintain a sustainable control of swine diseases, particularly those associated with PRDC pathogens.

## PRDC overview

PRDC is a multifactorial syndrome affecting the respiratory system of pigs in the swine industry worldwide (1, 2). In the PRDC-affected herds, substantial financial losses are related to the observed high percentages of mortality (2–20%) and morbidity (10–40%) (30), including the extra costs associated with the production failure and the associated medicinal / vaccination costs (31). During the last few decades, the causative agents of PRDC (including viruses, bacteria, and mycoplasmas) have been thoroughly investigated. The primary pathogens comprise of PRRSV, PCV2, IAV, ADV, and Mhp, acting as immunosuppressive or immunomodulatory agents in the host, while App attacks the phagocytic cells and impairs the defense mechanisms in the respiratory tract of the pig, thereby rendering it prone to secondary infections by other bacterial pathogens (such as Pm, Bb, Gps, and Ss) (1, 2).

Dynamic polymicrobial infections among the PRDC pathogens could lead to different clinical outcomes of coinfection or superinfection (2–6). A surveillance study of polymicrobial infections in weaning-to-finishing pig farms was monitored by using real-time PCR detection in oral fluid samples. The findings revealed an inconsistent detection pattern for PRRSV- and Mhp-induced infections between or within pens over time (32). The patterns and the levels of the PCV2 virus load in samples obtained from unaffected or affected farms by porcine circovirus-associated diseases (PCVADs) were different. A consistency of IAV detection was seen in clinical and subclinical cases as well as between pens at the same sampling timepoint, with the detection possible periods of 2–4 weeks during the IAV epidemic. This might be due to a continuous shedding of the IAV in the naïve population. Further findings revealed a good correlation of the PCR results obtained from multiple samples in the same pen, thereby suggesting that one should design the obtaining of samples from as many pens as possible, rather than focus on the collection of multiple samples from a limited number of pens. Using oral fluid sampling strategies in order to support on-farm investigations of respiratory diseases in pigs is recommended as a potential tool for disease survey, particularly for IAV (32). However, in the IAV endemic farms, the virus appears to circulate during the nursery period and within the gilt acclimatization period; therefore, the best samples for the IAV detection are samples obtained from those periods (33). Detection patterns for PRRSV, IAV and Mhp infections and their relationship to host immune responses could be used for design of appropriate management practices to control PRDC pathogens (32, 34, 35).

During the time before the ASF outbreaks, the PRDC situation in many countries appeared to be a major problem for the global pig industry (7). Major viral diseases (such as those caused by PRRSV and PCV2) played a pivotal role (with their high prevalence) in the disease complex. This situation dramatically changed after the ASF outbreak in Europe and Asia.

## Causative PRDC agents and their interaction

PRDC pathogens can interact with one another in a complex manner (36). Virus-to-virus, virus-to-bacterium, bacterium-to-bacterium, or other types of interactions are described. PRDC models are discussed below in terms of their effects on the clinical manifestation, the pathology of PRDC and their ability to alter the host immunity and immune response. Coinfection impact of major PRDC viruses in combination with other pathogens on disease severity, prevalence, and pathogenesis was summarized in Table 2.

**Table 2:** Coinfection impact of major PRDC viruses when combined with other pathogens on disease severity, prevalence, and pathogenesis.

**Virus**	**Impact on disease severity and prevalence**	**Impact on pathogenesis**	**References**
PRRSV	- Higher prevalence of App, Ss, and Gps following PRRSV infection. - Increased severity of clinical signs and Mhp-like pneumonia following an PRRSV infection or *vice versa*. - PRRSV infection was entirely hindered following an infection with App or App cell-free culture supernatant in SJPL and PAM cells. - Pigs that infected with PRRSV and *S. Choleraesuis* and received dexamethasone inducing stress had the most severe clinical signs, significantly longer and larger amount of *S. Choleraesuis* shedding in feces and PRRSV titers in sera.	- PRRSV is responsible for the worsening of the PCV2-related lesions and the enhancement of PCV2 DNA in the serum and of the PCV2 antigen in the tissue. - PRRSV and Mhp could damage the immune cells, thereby causing immunomodulation and triggering immunopathologies. - A pre-infection with PRRSV deteriorates the cytotoxic effects of bacteria on the cells.	(1, 37–42)
PCV2	- PCV2 and Ss2 coinfection induced more severe pneumonia, myocarditis, and arthritis, worsened the inflammatory response, and reduced the macrophage antigen presentation. - The severity of the PCV2-associated lung and lymphoid lesions, the amount of the PCV2 antigen, and the incidence of PMWS in pigs were worsen in the presence of Mhp.	- PCV2 promoted the App adhesion to PAMs during coinfection and suppressed the production of ROS by reducing cytomembrane NADPH oxidase activity, which was favorable for the *in vitro* survival of App in PAMs.	(1, 34, 43–47)
IAV	- The severity of the clinical disease, the lung lesions, the virus replication in lung, and the nasal shedding of the IAV increased because of a coinfection with App.	- Pre-infection with Mhp was followed by IAV (H1N1), in which the clinical outcome of the coinfection was aggravated with respect to the infiltration of phagocytic cells and the levels of proinflammatory cytokines	(48, 49)
ADV	- ADV may enhance the chances of a bacterial infection as well as the number of pathogenic strains in respiratory system of pigs, including Ss and avirulent strains of Gps, App, and Bb.		(50)

ADV, Aujeszky's disease virus; App, Actinobacillus pleuropneumoniae; Bb, Bordetella bronchiseptica; Gps, Gläesserella (Haemophilus) parasuis; IAV, influenza A virus; Mhp, Mycoplasma hyopneumoniae; NADPH, nicotinamide adenine dinucleotide phosphate; PAMs, porcine alveolar macrophages; PCV2, porcine circovirus type 2; PRDC, porcine respiratory disease complex; PRRSV, porcine reproductive and respiratory syndrome virus; ROS, reactive oxygen species; SJPL, St. Jude porcine lung (cells); Ss2, Streptococcus suis serotype 2.

### Virus-to-virus interactions and coinfections

PRRSV and PCV2 are the primary causative viral agents of PRDC (5, 30). Both PRRSV and PCV2 target the host immune defense by disrupting their immune function, thereby triggering an increased susceptibility to other pathogens that may affect the host growth performance sub-clinically and, in some cases, lead to lethality from associated diseases (31, 51). The interaction of PRRSV and PCV2 has been documented (6, 52, 53). The PRRSV is responsible for the worsening of the PCV2-related lesions and the enhancement of the PCV2 DNA in the serum and the PCV2 antigen in tissues (37). The understanding of the effects of PRDC agents on the host innate immune response is essential for the explanation of the virus-to-virus interactions. An experimental infection of pigs with IAV, PRRSV, or their combination revealed an induction of various local cytokine responses (54). Remarkably, a positive correlation was observed between the concentration of local tumor necrosis factor alpha (TNF-α) and interleukin-10 (IL-10) and the pig lung pathology, thereby suggesting that the cytokines were related to the lung pathological lesion induction. During infection, the local multiplication of both viruses also influenced the local cytokine response in the lung tissue. Strong associations between the local concentrations of TNF-α, interferon-gamma (IFN-γ), and IL-8 with the IAV (H1N1) and PRRSV antigen loads in the lung were observed. However, the coinfection or single infections had no significant effect on the lung pathology or the pathogen loading.

As far as the ASF pandemic is concerned, one of the major swine severe hemorrhagic diseases including the classical swine fever virus (CSFV) and the highly pathogenic PRRSV infections, the development of a multiplex real-time PCR exploring the presence of these agents in 1,143 specimens taken from various organs of dead pigs during the 2018–2021 period (55) revealed that the ASFV+CSFV, the ASFV+PRRSV, the CSFV+PRRSV, and the ASFV+CSFV+PRRSV coinfections were found in 2.45, 2.36, 1.57, and 0.17% of the examined cases, respectively. Although the interaction of those pathogens of interest was not assessed, the development of this assay certainly offered a tentative diagnosis of the major swine hemorrhagic diseases. In addition, a similar report revealed evidence of the ASFV+PCV2 coinfection (56). Recently, a comparison of the clinical and immunological responses to the highly virulent ASFV strain Armenia 2008 and the attenuated strain Estonia 2014 between specific pathogen free (SPF) pigs and commercial farm pigs vaccinated with PCV2, *Escherichia coli* and *Lawsonia intracellularis* vaccines were found to be diversified (57). The SPF pigs suffered to ASF disease after highly virulent strain inoculation much more than that of the farm pigs, whereas when infected with the moderately virulent Estonia 2014 strain, SPF pigs had shorter and lesser course of clinical symptom, compared to the commercial pigs. The author suggested that hygiene-related innate immune status might cause a double-edge sword impact on the immune responses to ASF. The higher baseline of innate immune activity supported reduction of initial ASF replication in the pig host, promoting the cytokine responses, and postponing lymphocyte proliferation following infection with the attenuated strain (57).

During the early period of the PCV2 discovery, a model of a well-known coinfection of PCV2, porcine parvovirus (PPV), and porcine cytomegalovirus was reported in the case of multiple abortions and reproductive failures (58). Coinfections of PCV2–4 and the novel parvoviruses PPV2–4 were detected through PCR in archival pig samples from the UK and neighboring countries. Evidence of the existence of PCV2 and PPV2-4 in clinically diseased pigs across production stages was also shown. Among the novel viruses (PPV2–7) identified since 2001, the novel PPV2 was the most dominant virus within the fattening age group. Moreover, based on statistical modeling through latent class analysis from clinical observation, pathology and laboratory findings, a clustering co-factor association between PPV2 and PCV2 was revealed (59). The results demonstrated that a novel PPV2 might involve in both the PRDC and the PCVADs.

### Virus-to-bacterium interactions and coinfections

In the early studies employing PRDC coinfection models during the 1990's, the capability of PRRSV to control the host immune response and, thereby, leading to the development of secondary bacterial infections was proposed. Nonetheless, as often observed under field conditions, the coinfection of a virus and a bacterium is found to be the main cause of PRDC, especially in cases of higher bacterial prevalence (60). The effects of the viral and bacterial coinfections can vary depending on their interactions. For example, the severity of an Mhp respiratory sign and pneumonia were found to be increased following a PRRSV infection. Similarly, after the Mhp infection, the PRRSV-induced disease and lesions were found to also worsen (38). As the years passed, more insightful studies on the interactions between viruses and bacteria have demonstrated that both the PRRSV and the Mhp can infect the immune cells or induce remarkable immunopathology. Both pathogens are also capable of altering the respiratory immune system, by increasing the susceptibility to other PRDC pathogens (1, 39). The apoptosis or the cell lysis of the pulmonary intravascular macrophages are induced during a PRRSV infection, thereby leading to an increase of the host susceptibility to bacterial agents such as Ss (39). *In vivo* experiments have confirmed that a coinfection of *Mycoplasma hyorhinis* (Mhr) and PRRSV can cause severe pathological lesions in the pig lung, and that these lesions are more extensive than those caused after an infection with Mhr or PRRSV alone (40). In 2014, a study using an *in vitro* mixed infection of PRRSV or IAV and App in a cell line, demonstrated the interactions between pathogens and host cells (41). The study showed that a pre-infection with PRRSV did not have an impact on the bacterial adherence to the cells, whereas a cytotoxic effect was additively taking place in this coinfection model. Interestingly, the PRRSV infection was entirely hindered when a pre-infection of St. Jude porcine lung (SJPL) cells and porcine alveolar macrophages (PAMs) with App or an App cell-free culture supernatant took place. Such an antiviral activity rather derived from small molecular weight, heat-resistant App metabolites (< 1 kDa), but not due to the lipopolysaccharide, was attributed (at least in part) to the production of IFN-γ. Another interaction model among PRRSV and *S. Choleraesuis*, and dexamethasone-induced stress in postweaning pigs was previously examined (42). Pigs dually infected with *S. Choleraesuis* and PRRSV showed clinical signs of dyspnea, and diarrhea with rough hair coat, whereas those received only PRRSV or *S. Choleraesuis* did not have obvious clinical signs. Interestingly, pigs received all 3 combinations had the most severe clinical signs, significantly longer and larger amount of *S. Choleraesuis* shedding in feces and higher PRRSV titers in sera. Therefore, this study demonstrated the synergistic interaction among PRRSV, *S. Choleraesuis*, and stress-induced by dexamethasone.

PCV2 is recognized as another major pathogen of PRDC (2). Coinfection of PCV2 with bacterial pathogen is frequently found in PRDC according to an earlier retrospective study from Korea (34). The authors reported that the prevalence of coinfections of PRDC pathogens were greater than those of single infections, and that Pm was the most prevalent bacterium found with PCV2 and Mhp. Opriessnig et al. have suggested that the severity of PCV2-associated lung and lymphoid tissue lesions, the amount and presence of the PCV2 antigen, and the incidence of PMWS in pigs were worsened in the presence of Mhp infection (43). In the past, an association between the Mhp vaccination and the PCV2 infection was controversial (44). An earlier study has demonstrated that the administration of Mhp bacterins shortly after an experimental PCV2 infection or a natural infection might enhance the severity of the PCV2-induced lesions and clinical signs of PMWS (45). To better understand the pathogenesis of the PCV2 coinfection, another study focusing on a PCV2 and Ss serotype 2 (SS2) coinfection has revealed that the severe pneumonia, myocarditis, and arthritis were induced due to a worsened inflammatory response and, probably, due to a reduced macrophage antigen presentation, thereby contributing to immune dysregulation and enhancement of the severity of the infection (46). Finally, a recent study has demonstrated that PCV2 can promote the App adhesion to PAMs during a coinfection and suppress the production of reactive oxygen species (ROS) by reducing the cytomembrane nicotinamide adenine dinucleotide phosphate (NADPH) oxidase activity; a development that is favorable for the *in vitro* survival of App in PAMs. During the infection, the PCV2 impaired the inflammatory response by decreasing the expression of TNF-α, IFN-γ, and IL-4 as well as the antigen presentation macrophages, thereby resulting in an impairment of the App clearance and a promotion of the App survival in PAMs (47). The immunosuppressive role of PCV2 is important in PCV2-systemic disease (PCV2-SD) in pigs (61). PCV2 infection can modulate the first immune response cells: macrophages and dendritic cells (DC). After engulfing and digesting by these cells, PCV2 does not replicate in conventional DC (cDC), whereas down-regulate the induction of interferon-α (IFN-α) by plasmacytoid DC (pDC). Production of interleukin-10 (IL-10) is also induced following PCV2 infection. The release of IL-10 induced by PCV2 infected peripheral blood monocytic cells (PBMC) suppresses the stimulation of IFN-γ, IFN-α and IL-12 production by the recall antigen of another virus. This may play an important immunosuppressive role of PCV2 pathogenesis (61–65).

Although Aujeszky's disease is a major swine viral disease that had been eradicated in several countries several decades ago, recent outbreaks of a new variant of ADV in China suggest that ADV might enhance the opportunity of bacterial infections (including those caused by Ss and avirulent strains of Gps, App and Bb) (50). Various detection rates of respiratory bacteria have been demonstrated among different specimen types since the selection of the sampling site is crucial for the determination for true disease prevalence. An evaluation of the dual infection by the IAV and the App has shown that the severity of the clinical disease, the lung lesions, the viral replication in the lung, and the nasal shedding of IAV were increased (48). Similar results were also obtained from another model of a pre-infection with Mhp followed by IAV (H1N1), in which the clinical outcome of the coinfection was aggravated in terms of the infiltration of phagocytic cells and proinflammatory cytokines (49).

Aspects related to environmental conditions, population size, management strategies, and pig-specific factors (including age and genetics) have been linked to the impact of the PRDC (2). The linkage of the pig age to the PRDC coinfection model has been documented by a previous study in which the prevalence of the PRDC pathogens was monitored through the undertaking of PCR in the oral fluid samples of 56 commercial swine farms in Korea. The rate of the PCV2 and Mhp PCR positivity were higher as the pig age increased, and the opposite was found to be the case with the Ss positivity (66). A disease monitoring study was recently undertaken in order to determine the prevalence of different PRDC pathogens and their seasonal variations in swine farms of Belgium and the Netherlands between 2011 and 2016, by using multiplex PCR (67). The analysis revealed that IAV, PRRS-European strain (PRRSV1) and Mhp were the major pathogens found during the weaning and the postweaning periods, while Mhp, PCV2, and PRRSV1 were the predominant pathogens identified in fattening pigs. Double infections were more prevalent than triple infections. Moreover, the prevalence of pathogens during the postweaning and fattening periods in the Winter were found to be higher than those of in Autumn. Climatological parameters (such as relative air humidity, air temperature difference, wind speed, wind direction, and duration of sunshine) might possibly have an impact on the prevalence of PRDC pathogens.

### Bacterium-to-bacterium interactions and coinfections

Coinfections of various bacteria located in the respiratory tract of pigs are often found in clinical cases. However, there is a certain difficulty in attempting to illustrate the interactions taking place within coinfections in *in vivo* models. The use of biofilm reflects the ability of bacteria to survive outside the host body (68), and their interaction may serve as a coinfection model. An *in vitro* model of the Ss and App coinfection was recently investigated under biofilm conditions (69), and revealed an upregulation of the App virulence genes, especially of the Apx toxin gene and the adhesin genes, as well as the induction of important virulence genes of Ss. The interactions of Ss and App might be responsible for the observed disease development and persistent infection. On the other hand, a model of ciliostasis induced by Bb could promote the adherence, colonization, and cytotoxic effects of a subsequent infection with the virulent strain of Ss serotype 2. It should be noted that the cytotoxic effect induced by Ss depended on the presence of suilysin (70).

## PRDC traditional management strategies in ASF-free countries or before the ASF introduction

During the last few decades, pig production has expanded from backyard farming to industrial enterprise. Basic farm management should be also capable of dealing with the rapid transformation on a more preventive basis; an approach generally known as “biosecurity.” To date, biosecurity measures have been widely implemented in livestock animal production worldwide. Theoretically, pig farms with low biosecurity tend to have higher chances of introducing and spreading diseases (71). Therefore, biosecurity must be improved so as to ensure appropriate farming conditions. The general concepts of biosecurity practice are mainly divided into two types: external and internal measures. External biosecurity measures include the physical barriers inhibiting the introduction of disease through the restriction of animal movement in the community, controlled movement of external semen sources, people, and vehicles, gilt and boar replacements and quarantines as well as the avoidance of any possible risks of disease transmission from the neighborhood, the feed, and the water (72, 73). Common internal biosecurity measures emphasize the spreading of a disease within the pig farm, and involve actions related to the farm management, personnel, facilities, cleaning, and disinfection (13, 73). The implementation of selected biosecurity programs is described below.

### Quarantine and acclimatization

In general, quarantine and acclimatization are considered as fundamental measures for the disease control and prevention strategies in swine breeding herds (74, 75). According to the dynamic of the breeding population of a given farm, appropriated replacement animals must be in place so that they become the future breeding stock, based on their performances for productivity, health, and profitability. However, replacement gilts are also a critical point of farm management, as many pathogens may be introduced if quarantine is improperly implemented. Therefore, disease monitoring must be emphasized in these populations to avoid disease introduction in breeding herds. In a former study investigating the introduction procedures of purchased breeding gilts in Belgian pig herds, suggested the adoption of quarantine and acclimatization protocols for gilts purchased and kept in the farm (internal quarantine), with a quarantine period of approximately 42 days (range: 14–140 days) and an acclimatization by employing feces from suckling piglets and placing them in contact with culled sows as a tool for the maintenance of optimal biosecurity (76). In theory, the acclimatization of the PRRSV can be accomplished by an introduction of naïve gilts to actively PRRSV-infected or -culled sows in a separated gilt preparation unit. Pathogen shedding from donor sows to recipient gilts through direct contact or by exposure to oral fluids or feces are thought to be mode of disease transmission or pathogen exposure. This could allow replacement gilts to become infected, recovered, and developed protective immunity to the farm pathogens before the breeding period. PRRSV vertical transmission is dramatically reduced by the proper gilt acclimatization demonstrating by negative weanling pigs. Nonetheless, oral fluids from donor sows may contain PRRS immunoglobulin (Ig). An association between the PRRS-specific IgA in oral fluids and the reduction of the PRRSV replication have been reported, thereby suggesting that the exposure to such oral fluids may control the PRRSV multiplication through the induction of signals that can reduce the macrophage susceptibility to the PRRSV infection (75).

### AIAO and batch production systems

Modern pig production using the AIAO production system has been introduced a few decades ago, by installing pigs under 1–2 weeks of age difference in the same environment (age segregation). This system potentially reduces the risk of disease transmission comparing to the traditional continuous production system, in which the younger animals might have exposed to endemic pathogens shedding from the older animals (77). The AIAO implementation improves the health and production outcomes of fattening pigs (78). The batch production system is operation-designed to improve the pig flow (by working with a group of sows and / or gilts with a 2-, 3-, 4-, or 5-week reproductive cycle as one batch). The most desired practice is a 3- or a 5-week batch. All animals in each batch are separated from other subsequent groups. Consequently, the AIAO system, the separation of age groups and the unidirectional flow are spontaneously operative and benefit the batch production system. The health status of the pigs and restriction of antimicrobial use are also improved (79, 80). In practice, most farms usually comply with management protocols comprising of several biosecurity measures at the same time. Recently, an evaluation of the PRRS stabilization protocols in 23 French farrow-to-finish farms located in a high-density swine area was conducted (81). The stabilization protocol included a mass vaccination using a modified live PRRS vaccine, herd closure, and strict internal biosecurity measures (including a unidirectional pig and human flow, as well as cleaning and disinfection). Such a protocol was able to stabilize most of the participating farms. The impact of the within-herd or internal biosecurity with the adoption of an AIAO and a batch-management system or room decontamination on the seroprevalence of *Salmonella* in slaughter pigs, was examined in a simulated analysis (82). An efficient room decontamination exerted the highest effect on the seroprevalence of *Salmonella* in pigs at slaughter, whereas only a slightly lower prevalence was observed as a consequence of the adoption of the batch production system. This investigation led to the conclusion that a good room decontamination with some flexibility with regard to pig mixing in the batch, are adequate in order to reduce the number of underweight pigs (82).

### Multisite production system and parity segregation

Disease outbreaks in pig production normally occur through vertical and horizontal transmissions. In order to control and prevent the opportunity of an outbreak, the design of the pig flow and the pig separation using multisite production and parity segregation is fundamental for the avoidance of disease transmission among different pig populations at risk, and can also improve the pig growth performances as a result (83). Multisite production management adopts the idea of age segregation to reduce the opportunity of pathogen transmission among sites (77, 84). Previous study revealed that prevalence of Mhp infection of the three-site systems was lower than that of one- or two-site system, suggesting that age difference of pigs might influence in Mhp transmission (77).

Parity segregation is the method used in order to separate pregnant gilts (parity 0; P0) and the first parity (P1) sows from second parity sows (P2) and above with respect to differences in their immune status, disease, nutritional management, and their offspring colostrum management. P1 sows and their offspring must be separated from P2+ sows (44). Ultimately, the benefit of using parity segregation is to gain better health and growth performance for the P1 sow-derived offspring. A reduction of disease transmission from P1 offspring to the offspring of other parity sows is, therefore, expected. Hence, parity segregation has been exploited as a measure for disease control in pig production, especially in cases of the PRRSV and the Mhp infection (44). Former reports on the prevalence of Mhp in breeding herds has shown that primiparous sows exhibited a higher prevalence of Mhp than multiparous sows (44, 85). This led to the suggestion of employing gilt vaccination, medication, and biosecurity measures, which might later have an impact on the Mhp prevalence in the sow herd.

Due to the massive impact of PRDC on the pig health, the production, and financial viability of the farm, the policy for the PRDC prevention and control through specific schemes for medication and vaccination may vary depending on the disease prevalence, the herd immunity status, and the biosecurity measures in place. In the practice under farm conditions in Thailand, gilts in the developing pool in Thai farms are generally twice-vaccinated during the age of 20–32 weeks with attenuated CSFV, ADV modified live virus (MLV), inactivated FMD, PRRSV MLV, PCV2, combined PPV-*Leptospira*-*Erysipelothrix*, and perhaps App vaccines in some endemic farms. Likewise, vaccinations for breeding herds are usually conducted through the mass vaccination of sows on production and comprise of 3–4 times per year administration of a live attenuated CSFV vaccine, an ADV MLV, an FMD-inactivated vaccine, and perhaps an PRRS MLV vaccine. For some PRRSV-stabilized sow herds, the PRRS type 1 inactivated vaccine may be implemented in pregnant sows if the farm is located within a PRRSV-endemic area (86, 87). Additionally, a PRRS MLV vaccine may be used for sow herd stabilization, as recommended by the Thai clinical practice guideline for the control of PRRS (88). However, the sow vaccination against Mhp is not necessary for the improvement of the Mhp colonization and the disease protection in piglets (89). Combination of vaccination and medication to control and prevent Mhp infection in piglet should also be considered (90, 91).

Since the antimicrobial resistance becomes an emerging problem threatening both the animal and the human health, the rational use of antimicrobials has been recommended. Based on data regarding the antimicrobial resistance prevention and control in agriculture and livestock animals, strategy 4 of Thailand's national strategic plan on antimicrobial resistance 2017–2022 has set a goal of a 20% and 30% reduction of antimicrobial consumption for humans and animals, respectively (92). Recently, the Department of Livestock Development (DLD) of Thailand has introduced regulations for the antimicrobial use in feed for disease prevention (prophylaxis) in commercial feed producers and farmers in 2018 and 2020, respectively. Since then, the use of beta-lactams, fluoroquinolones, polypeptide antibiotics, and fosfomycin in feed for disease prevention have been banned from the Thai livestock animal production. Several management strategies have been recommended in case of bacterial disease outbreaks. Antimicrobial use has been designed for different pig populations depending on the disease status. Principally, parenteral administration is suggested for sick pigs and pigs in close contact (93). The parenteral and / or oral administration of macrolides, aminocyclitols, tetracyclines, lincosamides, amphenicols, pleuromutilins and their combinations is primarily aiming for the treatment of Mhp infection (77, 94). In previous studies, antimicrobial treatment in feed following experimental Mhp infection could reduce the coughing sign and pulmonary lesion scores (91, 94). It should be noted that timing of infection, housing and management system, and strains of Mhp might have reflected on varied clinical sign development (95). Therefore, patterns of antimicrobial use for Mhp prevention and control should be aligned with the disease status and other related factors in the affected farms.

In summary, implementation of PRDC control and prevention measures mentioned above requires combination of several biosecurity measures of which the advantage of AIAO and batch production systems, pig and housing separations, quarantine and acclimatization, AIAO and, multisite production systems and parity segregation, and medication and vaccination strategies, *etc*. and should be considered based on a case-by-case basis depending on the disease prevalence, the herd immunity status, and the biosecurity measures. Improvement of biosecurity should be invested not only for PRDC control, but for ASF prevention.

## New normal management strategies after the ASF introduction

In recent years, ASF has triggered a remarkable crisis for pig production worldwide (7, 96). ASF is known as a human-driven disease (7). Transportation related to human activities and trading are considered as a major ASF transmission pathway (12, 22). In fact, the natural characteristics of the ASFV, especially the long-term survival of the ASFV outside the pig body and its resistance to environmental conditions (97, 98), can be particularly problematic for the success of ASF control in the endemic areas. Additionally, due to its large viral genetic size, its wide genetic variation, and its uncharacterized antigenic epitopes, the unclear mechanisms through with a protective immunity against ASFV can be achieved has delayed the successful vaccine development against ASF (99–104).

The first report of an ASF outbreak in China, the world largest pig farming country, was released in August 2018 (105, 106). Following the major outbreaks in China, the ASF has spread across East and Southeast Asian countries (96, 103, 107–112). Disease diagnosis in ASF-free countries has been mainly attempted based on clinical and laboratory monitoring *via* the use of real-time PCR (113–115). As far as the ASF prevention strategy is concerned, biosecurity measures are considered as the most effective methods, since the efficacy of developing ASF vaccines have not successfully met the expectancies (99, 103). Therefore, farm biosecurity improvement should be emphasized and should not be compromised in terms of the strict compliance to zero ASFV exposure. The Thai government has since then approved the preparation of emergency protocols at a national level through the cooperation among the DLD, the Ministry of Agriculture and Cooperatives, and other relevant agencies, swine farmers, and the private sector. It has also released the ASF contingency plans after the first ASF report in China. The preparedness of these plans are organized into “pre-outbreak,” “outbreak,” and “post-outbreak” periods, with an emphasis on the risk factors related to the control of ASF introduction (i.e., control of illegal movement of pigs and pork products across the border, restriction of tourists and visitors from ASF-affected countries, and control of fomites, pigs, food, and feed from ASF risk areas) (12).

In 2022, after hosting a workshop in Bangkok (Thailand), the Food and Agriculture Organization of the United Nations (FAO) and the World Organization for Animal Health (WOAH) have launched guidelines for the ASF prevention and control in smallholder pig farms in Asia (116). These guidelines described the ASF clean-chain system for smallholder pig farms, which are one of the pork value chain stakeholders in Asia. Ideally, biosecurity, risk surveillance, and risk identification / record keeping / traceability are defined as critical components for the implementation of risk mitigation measures. Additionally, the risk assessment of the ASF introduction and spreading must be accomplished together with the defining of the pork value chain and the establishment of a public-private partnership. In case of an ASF outbreak in the clean-chain area, the adoption of control measures, the investigation of the disease outbreak, as well as the diagnostic laboratory confirmation and reporting of the ASF-suspected cases should be achieved (116–118). In these guidelines, the recommendation of adopting quarantine measures (or segregated confinement) is considered as an important risk mitigation in order to decrease the probability of an ASFV introduction into the farm. All introduced pigs either from inside or outside the ASF clean-chain system are required to be quarantined and isolated at the source and / or at the target farm. A protocol of cleaning and disinfecting must also be strictly applied. During the quarantine period, the ASFV detection through PCR must be routinely performed (116).

A concern regarding the swill feed use by pig farms in Asia and Africa remains the major ASF prevention issue, as it might be a source of ASFV transmission (119). In 2020, an interview-based survey regarding farm management and biosecurity practices employed in order to fight the ASF outbreak and other domestic infectious diseases in 97 pig farms in Cameroon, revealed that the most important measures including the abandoning of swill feeding, as well as of the selling of sick pigs and community boar use, the avoiding of other livestock species, the pig barn fencing, and the enforcement of incoming stock quarantine should be implemented (120). Similar evidence has recently identified risks for the ASFV transmission in China (121). In the situations in which the swill feed use is unavoidable, one of possible ways to destroy the ASFV in swill is through heat inactivation. A recent study from Thailand has demonstrated a significant decrease of the ASFV titers in the swill at 70°C and 90°C after a 119- and 4-min heating, respectively (122). Interestingly, the findings differed from those upon previous FAO recommendations are based. Therefore, the prohibition of swill feeding in pig farms should be encouraged.

A spatiotemporal analysis was employed in order to describe the ASFV spread throughout Asian countries (including China, Mongolia, Korea, Vietnam, Laos, Cambodia, Myanmar, the Philippines, Hong Kong, Indonesia, Timor-Leste, and Papua New Guinea) from 1 August 2018 to 31 December 2019, and to provide an overview and a comparative analysis. The analysis revealed increased disease outbreak reports from China, Vietnam, and Laos representing two clusters of outbreaks, and further analysis indicated that the wild boar populations, the pig transportation, and the movement of contaminated fomites are probable risk factors for the ASFV spreading across Asia (107).

The Chinese and Vietnamese experiences in fighting the ASF outbreak were used as valuable knowledge for the forming of guidelines for other ASF-free countries and allowed them to prepare their own outbreak dealing plans. Following the identification and the confirmation of an ASF outbreak by veterinary and local authorities, the depopulation and the repopulation policies should be implemented together with a restriction of the human and pig movements (96). The FAO guidelines for the culling and the disposal have also been set in effect in 2022. The culling policy refers to both the planning of the culling operation and the culling methods themselves (123). In China and Vietnam, the culling of almost a whole operation unit, a site of the pig production, or a total depopulation (stamping-out) in order to avoid the spreading of ASF, was performed during the early stages of the outbreak, thereby causing a massive economic loss to the industry (124). In later stages, the culling policy through the tooth extraction protocol or a partial depopulation in both China and Vietnam has been modified with the adoption of more reasonable practices based on scientific evidence, thereby leading to a partial culling of sick pigs (21). The evaluation of the disease spreading pattern as well as the risk assessment regarding the intervention and the compliance of external and internal biosecurity measures, should be strictly done as a disease carrier may escape from the disease screening tool and may act in the future as a potential source of a new outbreak.

After the ASF introduction into Vietnam, the factors contributing to the overwhelming spread throughout the county include: (i) the poor biosecurity of the small and the small-to-medium commercial farms covering 80–90% of the farms in Vietnam, and (ii) the insufficiency of the veterinary services to deal with the outbreaks (125). Although there is no uniform definition of an ASF carrier in the surviving pig population, a previous review divided the surviving pigs into two main categories: (i) those that developed a persistent infection with periodic viremia and often show some clinical signs of subacute to chronic disease, and (ii) those that fully recovered after a shorter period of viremia leading healthy and productive lives (126, 127). Recent evidence suggests that ASF-recovering pigs are a potential source of reinfection since the virus remains in their tissues (128). It should be noted that infected–yet surviving–sows should not be further used for pig production, since the probability of stress (especially during the farrowing period) would induce the ASFV recurrence in the long-term survival pigs (25, 129).

The “rebirth” policy for pig farms in China and Vietnam, especially for small-to-medium pig holders, has been a topic of interest, particularly regarding the ASF re-occurrence due to biosecurity limitations. In an attempt to avoid the re-occurrence of ASF, the Chinese government and the related authorities have launched a program of financial support for culling a subsidy of the infected livestock, and have established the criteria for the repopulate farms (26), along with strict biosecurity management requirements for pig farmers, emphasizing the factors influence the rebirth of the pig farms provided in Table 3.

**Table 3:** Factors influencing the repopulation or rebirth of pig farms.

**Factors**	**Description**
Farm location	Repopulate farms in a new location with government support
Vehicles	Separate clean and disinfection bay for vehicles coming from outside the farm, with drying station; surface swab for PCR testing
Personnel / visitors	Quarantine for at least 72 h, showering, and disinfection
Fomites	UV or ozone treatment of equipment as well as of medicinal and biological products; swab sampling for PCR testing
Vectors / pests	Keeping free of birds, rats, flies, mosquitos, insects, and wildlife; routine use of pesticides
Food and water supply	Treating water supply with chlorine dioxide (3–5 ppm), and not allowing any pork or preserved pork consumption in the premise; sampling for PCR testing
Infrastructure	Cleaning and renovation by disinfection and painting; sampling for PCR testing
Pig house	Close system of pig house by using evaporative cooling system or air tunnel with a plastic net cover
Change of environment	Separate cement walkway from the vehicle road, with an optional disinfection bay
Sentinel pigs	Use weaned pigs as sentinel before moving fattening pigs
Replacement gilts / boars	PCR testing 2–3 times before bringing in; not allowing in any pigs that survived from the previous outbreak
Batch production / AIAO of farrowing unit	Calculate the batch of sows so as to fit the farrowing unit size, and generate an AIAO system; moving all sows in each batch to the farrowing unit on a single occasion; blood swab for PCR testing

AIAO, all-in all-out; h, hour; PCR, polymerase chain reaction; ppm, part per million; UV, ultraviolet.

Importantly, management strategies on how to reduce the human-to-pig and the pig-to-pig contacts should be planned so as to best fit the farm conditions. The use of combined viral and bacterial vaccines that are commercially available (such as PCV2 and Mhp), or the use of combined bacterial vaccines are effective alternatives (130). Intradermal / subcutaneous needleless vaccinations with an automatic injector have been used for vaccine and/or medicine administration and can substitute needle vaccination in the future. Lastly, concise vaccination programs should be designed. Vaccination against endemic diseases such as CSF, FMD, ADV, and PPV must be implemented, whereas vaccines for the prevention of other infectious diseases (including those triggered by PRRSV, PCV2, Mhp, and App) may be optional, depending on the farm disease status.

The ASF-induced farm disruption has caused the swine industry to rapidly transform itself, and to embrace the new era of swine farming. AI and smart farming have gained tremendous attention so as to resolve the challenges faced by intensive swine farming, and to enhance the productivity and the efficiency of the farms without compromising the strict biosecurity required during the ASF epidemic. The application of AI-assisted smart farming also provides essential information that can be utilized for diagnostic purposes, growth monitoring, and effective management (27–29). For example, environmental risk factors such as the ammonia concentration are known to be associated with respiratory health problems in finisher pigs, and can be detected through electrochemical sensors, thereby assisting the decision-making management strategies in the farm (28).

**Figure 1:**
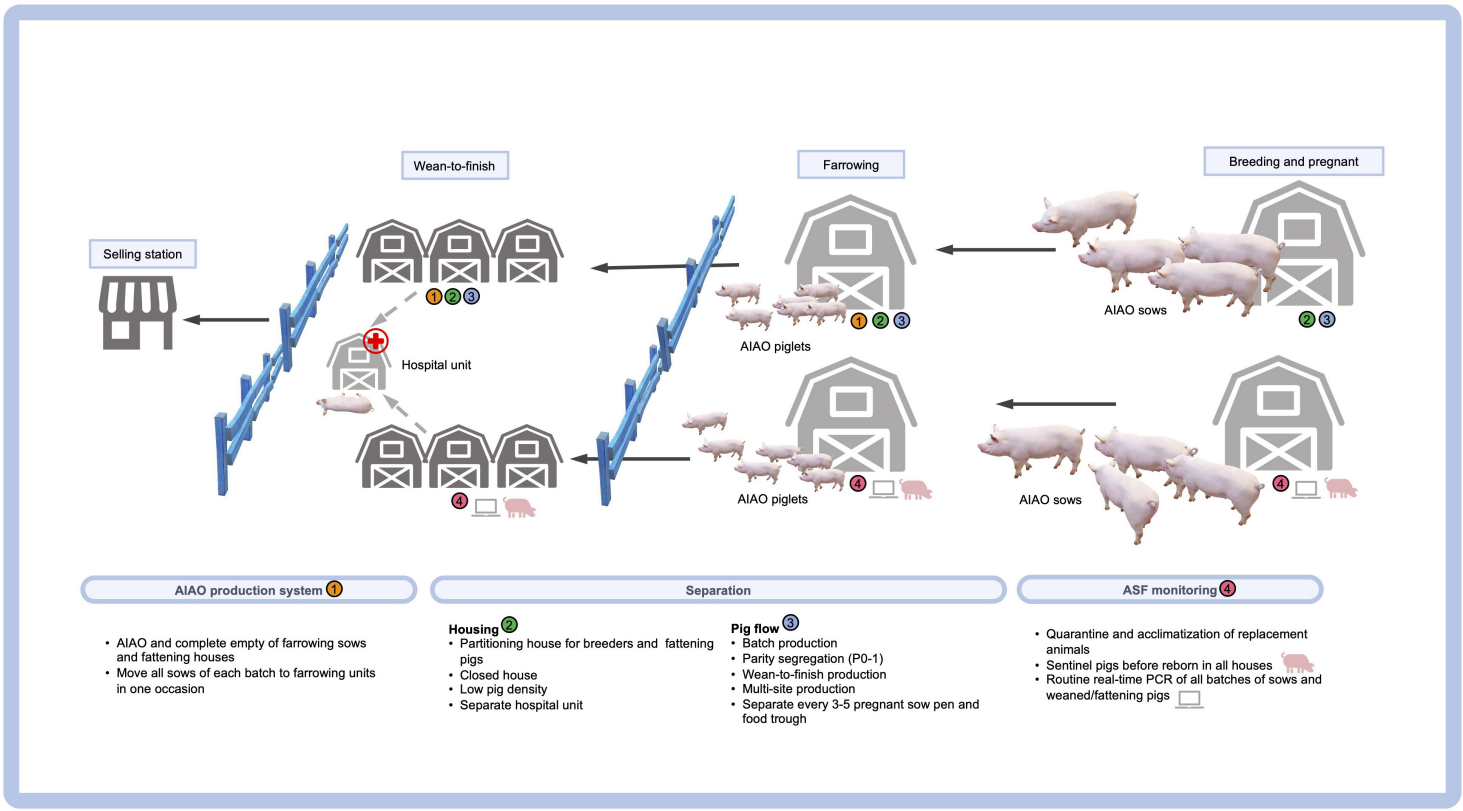
Internal biosecurity measures regarding the pig flow in a “repopulate” farm.

The new normal biosecurity required for ASF-affected areas has introduced many promising tools in the ASF-affected countries. Apart from the external biosecurity management, a checklist for internal biosecurity would routinely ensure the confining of the entry zone, applying quarantine measures in the buffer zone, generating a clean zone, and increasing hygiene and awareness among the farm workers (12). A list of internal biosecurity measures regarding the pig flow for the repopulate farms is provided in Figure 1. It should be noted that the routes of transportation of the carcass to slaughter is also a potential source of disease spreading in the affected areas and could therefore contaminate the pork production chain. After the farm repopulation, the herd biosecurity should be strictly followed, while regular PCR monitoring should be enforced when pigs are sick or found dead. The new normal management practice for repopulate farms located in a neighboring location (farm zoning) also requires the adoption of a partitioning strategy. A combination of all aforementioned biosecurity strategies is essential for the sustainable control and prevention of ASF and will eventually eliminate some swine diseases (but not PCV2). This is due to previous studies that provided evidence of high levels of PCV2 DNA not only in animals, but also in the farm personnel and the environment (including the offices), thereby indicating a new and long-lasting possible source of a within-the-farm transmission of PCV2 (131, 132).

Based on our observations regarding the ASF-survived and repopulate farms, the PRDC-associated problems seem to have disappeared or to have not affected the production performance, possibly due to the fact that most of the pathogens were eradicated after the ASF-induced depopulation together with pathogen reduction from the current strict biosecurity. However, in some farms, clinical signs related to PCV2 are rising after the abandoning of the PCV2 vaccination (so as to avoid human-to-pig contact) during the ASF epidemic (133). It should be noted that the PCV2 vaccination should still be recommended in the PCV2-endemic farms (134, 135).

## Conclusions

PRDC is a major multifactorial disease affecting the respiratory system of pigs in the swine industry worldwide. Dynamic polymicrobial infections triggered by PRDC pathogens have clinical outcomes that depend on the type of coinfection occurring each time. Traditional PRDC management strategies (including quarantine and acclimatization, AIAO and batch production systems, multisite production systems and parity segregation, medication, and vaccination programs, *etc*.) are implemented based on a case-by-case basis depending on each farm's health status. After the ASF outbreaks, the adoption of new normal management strategies minimizing pig contact to lessen the opportunity of human-to-pig contact and reducing the number of vaccines administered more or less may lead to a re-emergence of existing endemic PRDC-causing pathogens in case of endemic PCV2-infected farms. Vaccination is essential for the endemic pathogens, but increased numbers of pig vaccination may lead to increased risks of ASF transmission. To deliver clear and concise vaccine recommendation, the farm veterinarians should be responsible for the vaccination program based on the farm disease status. Another good scenario after the ASF-induced depopulation is that the concise pig vaccination program and change of biosecurity may help simultaneously eliminate PRDC from the farms. The disruption caused by ASF has forced the swine industry to rapidly embrace a new era of swine farming. AI and smart farming have gained tremendous attention in order to resolve challenges in intensive swine farming. They have managed to enhance the farms' productivity and efficiency, despite the strict biosecurity required during the ASF era.

## Author contributions

PA and RT contributed to conception and design of the review article. PA organized the database and wrote the first draft of the manuscript. Both authors contributed to manuscript revision, read, and approved the submitted version.

## Funding

This work was funded by the National Research Council of Thailand (NRCT): RT NRCT Senior Scholar 2022; Grant Number N42A650553.

## Conflict of interest

The authors declare that the research was conducted in the absence of any commercial or financial relationships that could be construed as a potential conflict of interest.

## Publisher's note

All claims expressed in this article are solely those of the authors and do not necessarily represent those of their affiliated organizations, or those of the publisher, the editors and the reviewers. Any product that may be evaluated in this article, or claim that may be made by its manufacturer, is not guaranteed or endorsed by the publisher.
